# Impact of *Bifidobacterium infantis* supplementation on growth, health outcomes, and gut microbiome features in underweight infants from Pakistan

**DOI:** 10.3389/fnut.2026.1783141

**Published:** 2026-04-29

**Authors:** Janie Parrino, Justine Sunshine, Kenneth Tripp, Michael Shaffer, Ume Sughra, Nicola Procházková, Marlene Jara, Janne Marie Moll, Robert Noble, Lori Muir, Emily McIntyre, Elif Guduk, Devika Zachariah, Cecile Vernochet, Nicole Frahm, Alexander C. Schmidt

**Affiliations:** 1 Gates Medical Research Institute, Clinical Development, Cambridge, MA, United States; 2 Al Shifa Research Centre, Rawalpindi, Punjab, Pakistan; 3 Cmbio, Copenhagen, Denmark

**Keywords:** *Bifidobacterium infantis*, biomarkers, malnutrition, metabolomics, metagenomics, microbiome, probiotic

## Abstract

**Background:**

Alterations in the gut microbiome are implicated in infant malnutrition. *Bifidobacterium longum* subspecies *infantis* (*B. infantis*), a commensal common in breastfed infants, has been shown to have reduced abundance in malnourished infants. This trial (NCT05952076) evaluated if *B. infantis* strain Bi-26 supplementation could improve growth and health outcomes in underweight infants in Pakistan.

**Methods:**

In this double-blind, randomized, placebo-controlled trial, 40 infants aged 30–120 days (d) with a weight-for-age Z score (WAZ) below −2 received daily oral Bi-26 or placebo for 28d, with follow-up to d90 for safety. The primary endpoint was change in WAZ from baseline to d56. The intended sample size was 396 infants but study was terminated early due to operational delays. Total *B. infantis levels* microbiome, metabolome, and cytokine profiles were assessed.

**Results:**

Bi-26 supplementation increased fecal *B. infantis* levels at d28 (*p* = 0.001) and d56 (*p* = 0.03) but did not result in significant change in WAZ (*p* = 0.69) or weight gain (*p* = 0.56) compared to placebo. Fewer adverse events (AEs) occurred in the Bi-26 group compared to placebo (40% vs. 80% of infants; 17 vs. 49 events). Probiotic engraftment was impacted by presence of baseline endogenous *B. infantis*, suggesting that Bi-26 complemented rather than outcompeted endogenous strains. Bi-26 altered microbiome composition with transient alterations in function and metabolite abundance that reverted to baseline by d56, without cytokine differences between groups. *B. infantis* levels and Bifidobacterium-community types were associated with fewer AEs but not changes in WAZ or weight.

**Discussion:**

Bi-26 supplementation had an acceptable safety profile but did not improve growth. The findings of this trial support further evaluation of *B. infantis* strains in larger studies of underweight infants across diverse LMIC settings. Future trials should determine whether sustained metabolic and functional remodeling can translate into measurable improvements in growth and health outcomes.

**Clinical trial registration:**

https://www.clinicaltrials.gov/study/NCT05952076, NCT05952076.

## 1 Introduction

Undernutrition contributes to around 45% of deaths among children under 5 years of age, most commonly in low- and middle-income countries (LMICs) (1). A healthy infant gut microbiome contributes to digestive metabolic balance and systemic physiological functions that are essential to infant growth and development, including development of the immune system, metabolism, and colonization resistance to enteric pathogens (2). Gut dysbiosis, an imbalance in microbial composition characterized by the overrepresentation of potentially pathogenic taxa, is implicated in mediating persistent pathophysiological and immune abnormalities. It is thought to play a role in nutrient malabsorption in infants leading to lowered weight and failure to thrive (3–5).

*Bifidobacterium longum* subspecies *infantis* (*B. infantis*) is a commensal bacterial strain that is commonly found in the microbiome of breastfed infants younger than 6 months of age in LMICs (6, 7). *B. infantis* possesses multiple conserved gene clusters for human milk oligosaccharide (HMO) utilization, allowing for efficient import and intracellular degradation of diverse HMOs (6, 8). *B. infantis* colonization has been associated with host benefits, including reduced pathogen burden, accelerated immune maturation, decreased gut inflammation, improved epithelial barrier integrity, and greater production of health-promoting short-chain fatty acids (SCFA) (6, 9–11).

*Bifidobacterium infantis* has been reported to have decreased abundance or be absent in infants who are severely undernourished relative to controls (12). In the SYNERGIE pilot trial in Bangladesh, hospitalized 2–6-month-old infants with severe acute malnutrition (SAM) who received daily *B. infantis* EVC001, with or without a prebiotic, for 4 weeks demonstrated successful colonization and greater gains in weight-for-age z score (WAZ) versus placebo (12). Importantly, SYNERGIE evaluated a single strain, leaving open the question whether other *B. infantis* strains may result in similar benefits and whether strain-agnostic supplementation could be used to support recovery. To address this gap, the CONSTELLATION trial was designed to build on the findings from the SYNERGIE trial and assessed a different *B. infantis* dietary supplement, Bi-26, on growth outcomes. The CONSTELLATION trial also included exploratory analyses to evaluate the mechanistic hypothesis that *B. infantis* supplementation promotes nutritional recovery and growth by correcting gut dysbiosis and through downstream effects on immune and intestinal barrier function (9). Here, we report the trial’s primary outcomes alongside these biomarker findings.

## 2 Methods

### 2.1 Clinical trial registration

The study’s first participant was enrolled on July 3, 2023, and the trial was registered at ClinicalTrials.gov on July 11, 2023, 8 days after initial enrollment. This timing is compliant with the ClinicalTrials.gov Protocol Registration and Results System (PRS) requirements established under FDAAA 801 and the Final Rule (42 CFR Part 11). Please refer to FDAAA 801 and the Final Rule | ClinicalTrials.gov for the full name of the referenced PRS guidelines and NCT05952076; https://www.clinicaltrials.gov/study/NCT05952076 for the clinical trial registration records.

### 2.2 Study design and participants

**Figure 1:**
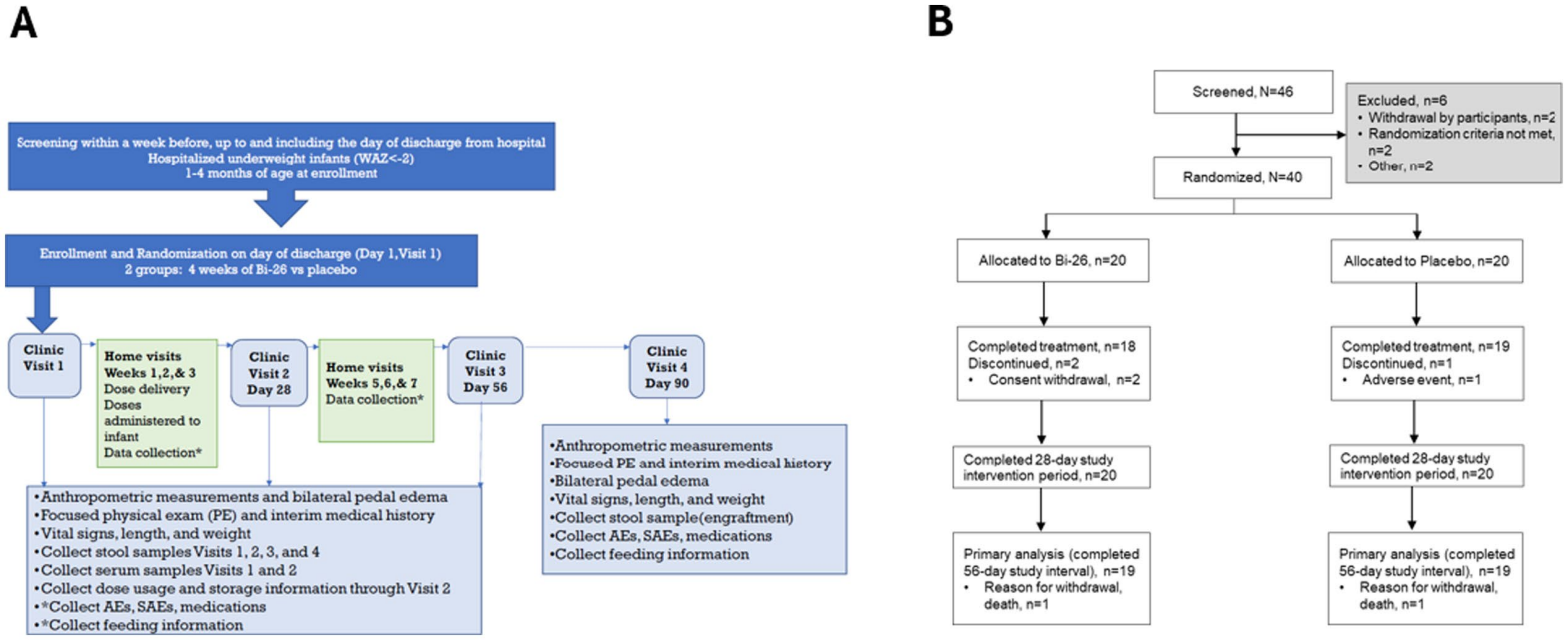
CONSTELLATION trial design and CONSORT diagram. **(A)** On day 1 (day of hospital discharge, before supplementation), infants hospitalized for acute nonsurgical illness were randomized to receive a single dose of Bi-26 or placebo each day for 28 days. Infants were followed through clinical and home visits for a total of 90 days. **(B)** Screening, randomization, and completion of treatment, study intervention period, and primary analysis period (d1–d56).

CONSTELLATION was a randomized, double-blind, placebo-controlled trial (Figure 1A) conducted at five sites in Pakistan. Infants aged 30–120 days with a WAZ below −2 were enrolled upon hospital discharge following an acute, non-surgical illness. Eligibility required completion of any fluid or antibiotic treatment before enrollment and at least some ongoing and intended breastfeeding. Infants were excluded for congenital disorders affecting feeding or growth, prolonged hospitalization since birth, or failure to be discharged from hospital for at least 1 week. Additional exclusions included septic shock, mechanical ventilation, acute kidney injury, severe jaundice or suspected kernicterus, tuberculosis, or HIV exposure or infection. Ongoing antibiotic or probiotic use in the infant or mother were exclusionary.

Participants were randomized to receive daily Bi-26 (IFF Health and Biosciences, Danisco USA, Inc) or placebo for 28 days, with follow-up to day 90 (d90). The trial was designed as a multi-country Phase 3 study targeting enrolment of 396 infants in LMICs but was terminated early due to operational delays following a temporary enrolment suspension in Pakistan. The suspension, requested by the Drug Regulatory Authority of Pakistan and the National Bioethics Committee to review safety, led to an independent IDMC assessment. Although the IDMC recommended that the trial continue without modifications, the suspension of enrolment was not lifted. The Sponsor, therefore, elected to discontinue the trial.

### 2.3 Randomization and masking

At hospital discharge (d1), participants were randomized 1:1 to receive Bi-26 or placebo using a centralized Interactive Web Response System. Randomization was stratified by age at enrollment (30–59 days or 60–120 days), discharge destination (home or nutritional rehabilitation unit), and WAZ (≤ − 3 vs. > −3) to balance groups across key factors. Placebo doses were matched to Bi-26 in appearance, odor, packaging, and fill weight to maintain blinding.

### 2.4 Procedures

Bi-26 or placebo was supplied as a powder to be mixed with 3–5 mL of breastmilk and administered daily by oral syringe. Following WHO guidance favoring exclusive breastfeeding for the first 6 months, breastmilk was preferred; if unavailable, the dose could be mixed with safe water. Each Bi-26 sachet contained approximately 5 billion colony forming units (CFU) of *B. infantis* at the end of shelf life with potato maltodextrin as excipient; placebo contained maltodextrin only. Caregivers received weekly sachets for 4 weeks, stored refrigerated or in provided cooler bags if electricity was unreliable.

The first dose was given post hospital discharge. Weekly visits continued through d56 (clinic on d28 and d56; otherwise home), with a final clinic visit on d90. Adverse events and serious adverse events were recorded through d90, and efficacy assessments were performed at clinic visits. Standardized scales and infantometers were used across sites to assess weight and length. Fecal samples were collected on d1 (before dosing), d28, d56, and d90 for engraftment and exploratory analyses. Feeding history was documented by caregivers throughout the trial.

### 2.5 qPCR assay and clinical testing

The qPCR assay targets an intergenic region near the phosphate acetyltransferase gene of Bi-26 and the intended use of this assay is to enable accurate quantification of *B. infantis* Bi-26 levels in fecal samples collected in clinical research settings as previously described (13). Bioinformatic analysis against public *B. infantis* sequences indicated that the primers matched the vast majority of known *B. infantis* strains (data not shown) and were therefore used to quantify total *B. infantis* levels in this study. Fecal samples were collected in OMNIgene OM-200 tubes and sent to Wisplinghoff Laboratory (Cologne, Germany). DNA was extracted within 45 days using the ZymoBIOMIC 96 MagBead DNA kit per manufacturer protocol. When samples were viscous or lacked sufficient stabilizing liquid, OMNIgene Liquefaction Reagent was added. DNA yield was quantified with the Qubit dsDNA HS Assay Kit and purity assessed by A260/A280 ratio on a NanoDrop spectrophotometer. Extracts were stored at −80 °C until analysis, with extraction controls assessed by qPCR monthly. All samples from an individual were processed on the same plate. Five μL of DNA eluate (diluted 1:10 if ≥ 2 ng/μL) was added to a 25 μL qPCR reaction. Plates included validation standards, no-template and positive controls (purified Bi-26 DNA), and quality-control wells. Samples were run in singlet on 96-well plates. Quantitative abundance was calculated as previously described (13). Results above the lower limit of quantification (LLOQ) were reported as positive (quantified in genomes/ng DNA) and below LLOQ as negative.

### 2.6 Metagenomic sequencing and data processing

Fecal DNA extracts (described above) from clinical stool samples collected at Days 1, 28, and 56 were shipped on dry ice to Clinical Microbiomics (Cmbio, Copenhagen, Denmark). DNA libraries were prepared using the Watchmaker DNA Library Prep Kit (7K0019-1K) and the Twist Universal Adapter System. Genomic DNA was enzymatically fragmented using the Watchmaker Frag/AT Buffer and Enzyme Mix to generate fragment sizes appropriate for paired-end sequencing. Twist Universal Adapters (10×) and Twist HT Unique Dual Indexes (2×) were ligated to the DNA fragments, followed by seven PCR amplification cycles to enrich adapter-ligated products. The libraries were purified using AMPure magnetic beads (Beckman Coulter) and eluted in nuclease-free water. Library concentrations were quantified using the Qubit dsDNA High Sensitivity Assay Kit (Thermo Fisher Scientific), and pooled libraries were sequenced on an Illumina NovaSeq X platform to produce 2 × 150 bp paired-end reads with an average sequencing depth of 34.2 million read pairs per sample (range: 15.5–84.1 million). All samples passed internal quality thresholds, yielding consistent sequencing depth and low host contamination.

Raw FASTQ files were processed through a standardized quality-control pipeline. Reads mapping to the human reference genome (GRCh38) were removed using Bowtie2 (v2.4.2), and adapters and bases with Phred scores below 30 were trimmed with AdapterRemoval (v2.3.1). Read pairs passing these filters with a minimum read length of 100 bp were classified as high-quality non-host (HQNH) reads, accounting for approximately 94% of total reads per sample.

### 2.7 Stool metabolomics clinical testing

The following metabolites were assessed: short-chain fatty acids (SCFA) panel (Acetic acid, Butanoic acid, Heptanoic acid, Hexanoic acid, Pentanoic acid, 2-methylpropanoic acid, i3-Methylbutanoic acid, Formic acid, Propanoic acid, 4-Methylpentanoic acid,) and broad Tryptophan panel Alanine, 2,3-Pyridinedicarboxylic acid, 2-Oxoglutaric acid, 2-Picolinic acid, 3-(4-hydroxyphenyl)propionic acid, 3-Methylindole, 5-Aminovaleric acid, Anthranilic acid, Asparagine, Aspartic acid, cis-Aconitic acid, Citric acid, Cystine, Fumaric acid, Glutamic acid, Glutamine, Glycine, Histidine, Indole, Indole-3-acetic acid, Indole-3-acrylic acid, Indole-3-butyric acid, Indole-3-propionic acid, Indole-3-pyruvic acid, Isocitric acid, Isoleucine, Lactic acid, Leucine, Lysine, Malic acid, Methionine, Ornithine, Phenylalanine, Phosphoenolpyruvic acid, Proline, Serine, Succinic acid, Threonine, Tryptamine, Tryptophan, Tyrosine, and Valine.

Fecal material was homogenized in ultrapure water containing internal standards using a bead beater for 4–6 min. After centrifugation to pellet non-soluble material, the supernatant was filtered through 0.22 μm centrifuge filters. For SCFA analysis, samples were acidified with hydrochloric acid and spiked with isotope-labelled standards. For the tryptophan-related metabolite panel, samples were fortified with isotope-labelled standards, derivatized with methyl chloroformate (MCF) to form volatile derivatives, and purified via two-phase extraction before gas chromatography–mass spectrometry (GC–MS) analysis.

Both SCFA and tryptophan panels were analyzed using Agilent 7890B GC coupled to a 5977B quadrupole mass spectrometer with a high-polarity ZB-FFAP column (30 m × 0.25 mm × 0.25 μm). Data were acquired and processed using ChemStation and Skyline software. Compound quantification was performed against external calibration curves using in-house software. Quality control (QC) samples were prepared by pooling equal aliquots from all samples and analyzed after every sixth study sample to monitor drift and matrix effects.

### 2.8 Stool cytokine panel assay validation and clinical testing

An electrochemiluminescence (ECL)-based assay based on the Meso Scale Discovery (MSD) V-PLEX Proinflammatory Panel 1 (human) kit, was developed and validated by Synexa Life Sciences to quantify 10 proinflammatory cytokines (IFN-*γ*, IL-1β, IL-2, IL-4, IL-6, IL-8, IL-10, IL-12p70, IL-13, and TNF) in human stool lysates. Key parameters assessed included accuracy, precision, selectivity, parallelism, and sample stability. Validation samples comprised calibration standards, quality control (QC) materials, and endogenous stool samples derived from both adult and infant donors to ensure matrix suitability for the intended context of use. Quantitation was based on a four-parameter logistic regression model with 1/Y^2^ weighting, and predefined acceptance criteria required coefficients of variation (%CV) and relative error (%RE) within 20% (≤25% at the lower and upper limits of quantification) for calibration controls and within <30% for QC controls. Method performance was evaluated across multiple independent assays runs to assess intra- and inter-assay reproducibility. Parallelism studies were conducted to confirm comparable dilution-response relationships between calibration standards and endogenous matrices, ensuring accurate quantification in stool lysate. Stability assessments examined sample integrity under representative conditions, including short-term (on-bench) stability at room temperature or 2–8 °C for up to 4 h and long-term freeze–thaw stability for up to four cycles at −80 °C.

Stool samples were shipped on dry ice to Synexa Life Sciences (Cape Town, South Africa) and stored at −80 °C until analysis. On the day of testing, samples were thawed at 2–8 °C and diluted 1:2 (50%) with assay diluent prior to analysis. For stool samples, approximately 100 mg aliquots were homogenized in lysis buffer to generate a lysate at 142.9 mg/mL, vortexed to ensure uniformity, and centrifuged at 700×*g* for 10 min at 2–8 °C to remove insoluble material. The resulting supernatant was aliquoted into single-use tubes and maintained at low temperature until use. Stool matrices were analyzed using validated method described above. Each plate included calibration standards, quality control samples, blanks, and study samples, with all samples analyzed in duplicate wells. Following incubation with SULFO-TAG™ detection antibodies, plates were developed using MSD Read Buffer T and read on an MSD Sector Imager S600.

### 2.9 Outcome assessments

The primary objective was to evaluate change in WAZ from baseline to d56. The key secondary objective assessed change in absolute weight (grams) over the same period. Additional secondary objectives included treatment response over time, the proportion (percentage) of infants achieving predefined WAZ improvements or no longer meeting criteria for underweight at d56, and re-hospitalization rates. Safety was evaluated throughout the study by monitoring adverse events and serious adverse events. Fecal *B. infantis* levels (genomes/ng) were quantified over time to assess engraftment.

### 2.10 Statistical analysis

The planned sample size of 396 infants (198 per group) was determined pragmatically to balance demonstration of a clinically relevant outcome with feasibility considerations. At the initial planned sample size, the study was powered to detect a 0.45 difference in mean change in WAZ (SD 1.3) between Bi-26 and placebo with 90% power and two-sided *α* = 0.05. However, enrollment stopped early after 40 participants were randomized.

For the primary analysis, data from participants randomized to the Bi-26 group were compared to data from participants randomized to placebo. For continuous endpoints, a mixed model for repeated measures (MMRM) was used to estimate the Least Squares Means (LS Means) difference in change from baseline between Bi-26 and Placebo. The MMRM had fixed effects for treatment, visit (categorical), treatment*visit interaction, WAZ (continuous) and age (in days) at baseline, country, and discharge destination as covariates. The LS Means estimate the means for each treatment after adjusting for the effects and covariates in the model. For the binary response outcomes, the proportion of success, and the associated 95% confidence interval were calculated using the mid-P method.

The modified intention-to-treat (mITT) population included all randomized participants receiving ≥1 dose; the per-protocol (PP) population included those completing d90 without major deviations; and the safety population included all who received any dose. For the mITT population, participants were analyzed according to the intervention to which they were randomized. For the PP and safety populations, participants were analyzed according to the intervention actually received. All stats analyses were performed using SAS® code. This study is registered on ClinicalTrials.gov (NCT 05952076) and PACTR (24166).

### 2.11 Exploratory analyses

All exploratory laboratory assessments and analyses were conducted using the mITT population.

### 2.12 qPCR

Quantitative *B. infantis* abundance was compared between groups using the Mann–Whitney *U* test, and correlations with WAZ, weight, and total adverse events through d90 were evaluated using Spearman’s rho (performed in SciPy v1.13.1).

### 2.13 Metagenomics

Taxonomic profiling was performed using the Clinical Microbiomics proprietary CHAMP™ pipeline to generate relative abundance profiles across multiple taxonomic levels (species to phylum) (14) using the Clinical Microbiomics Human Microbiome Reference (HMR05) gene catalog, which comprises 25.8 million genes derived from >30,000 human microbiome samples spanning 6,567 prokaryotic and 244 eukaryotic species. HQNH read alignment to the catalog was performed using BWA-mem (v0.7.17), retaining only reads with ≥95% identity and mapping quality (MAPQ) ≥ 20. Species-level abundance estimation relied on up to 250 species-unique signature genes per species, selected for high prevalence (≥60%) and species specificity (14). Read counts were modeled using a negative binomial distribution normalized by effective gene length and adjusted for dispersion, with final abundances normalized to sum to 100% per sample.

Functional annotation of prokaryotic genes was conducted using EggNOG-mapper (v2.1.7) in DIAMOND mode, assigning orthologous groups and KEGG Orthology (KO) identifiers. Eukaryotic genes were annotated using KofamScan, and higher-level pathway summaries were generated for KEGG modules, Gut Metabolic Modules (GMMs), Gut-Brain Modules (GBMs), and Human Milk Oligosaccharide (HMO)-specific Carbohydrate-Active Enzymes (CAZymes). Functional potential profiling was based on mapping annotated genes to KEGG Orthology (KO) identifiers and aggregating them into KEGG modules (v78.2) to infer microbial metabolic capacity. Custom curation expanded the functional database to include fucose and sialic acid degradation pathways characteristic of *Bifidobacterium* metabolism. Functional modules were considered present in a species if ≥2/3 of the required enzymatic steps were detected, or all steps were present for pathways with ≤3 reactions. Module abundances were calculated by summing the relative abundances of all contributing species, with both raw and rarefied relative abundances used for downstream testing.

Species relative abundances were aggregated to genus, family, order, class, and phylum levels for visualization and group comparison. The final dataset included 1,022 detected species across all samples, with an average of 69 species per infant.

Alpha and beta diversity were estimated from rarefied species abundance matrices. Alpha diversity was assessed as both *species richness* (number of observed species) and *Shannon diversity index* (accounting for evenness of species abundance). Beta diversity, reflecting compositional dissimilarity between samples, was calculated using the Bray–Curtis dissimilarity index and visualized via Principal Coordinates Analysis (PCoA). Group-level differences in community composition were tested using Permutational Multivariate Analysis of Variance (PERMANOVA) implemented in the *vegan* R package (adonis2 function, 1,000 permutations). Differences in alpha diversity, taxon abundance, and prevalence were analyzed cross-sectionally at each timepoint using a linear mixed model of the form *feature ~ intervention + (1|site_number)*. *p*-values were adjusted for multiple comparisons using the Benjamini–Hochberg false discovery rate (FDR) procedure, with statistical significance defined as nominal *p* < 0.05 or FDR-adjusted *p* < 0.10.

To explore global microbiome structure, Fecal Community Types (FCTs) were defined using Dirichlet Multinomial Mixture (DMM) modeling based on species-level abundance profiles. Models were fitted with 1–10 Dirichlet components and evaluated across 10 iterations using Laplace approximation, Akaike information criterion (AIC), and Bayesian information criterion (BIC) to identify the optimal number of clusters. Three FCTs were ultimately selected: FCT1 (dominated by *B. infantis*), FCT2 (characterized by *E. coli*, *B. breve*, and *B. bifidum*), and FCT3 (dominated by *B. breve*). Transitions in FCT clusters over time were visualized to assess microbiome trajectory and intervention effects, with differences in cluster distribution tested by chi-squared analysis.

### 2.14 Strain tracking

Sample sequencing reads were mapped to the species-specific signature genes for the CHAMP species. Single nucleotide variants (SNVs) at each position of the *B. infantis* signature genes optimized for accurate abundance profiling were identified using BCFtools multiallelic-caller (v.1.18) (15, 16). In addition to the study samples, simulated error-free 150 bp paired-end reads from the reference strain genomes were generated using ART (v. 20,160,605) and processed using the same workflow as described for the study samples to generate strain-specific SNV profiles.

SNV profiles were generated from species-specific signature genes. Signature genes identified as outliers in >50% of samples (based on 99% quantile thresholds from species relative abundance) were removed from SNV-level analyses. Mixed positions were defined as sites with ≥2 alleles, and polymorphic positions as sites distinguishing strains. Baseline (endogenous) strains were extracted when ≥33% of signature genes were covered at depth ≥2, the dominant allele frequency was ≥95, and <0.5% of sites were mixed. Positions with depth >2 standard deviations above the median were removed as outliers.

If an endogenous baseline strain was present, strain abundances were estimated from the median depth at polymorphic positions where the endogenous strain differed from the reference strain. If an endogenous baseline strain was not present, strains were inferred as follows: When minor alleles had depth >1, two Gaussian 2-component mixture models (one for alleles matching the reference, one for alleles not matching it) were fitted to allele depth versus total depth. Low-depth unexpected alleles were treated as sequencing noise, and strain abundances were derived from the median depth of non-noise positions. If mixture models failed or all minor alleles had depth = 1, a fallback procedure was applied: Samples with <10,000 covered positions were classified as undetermined, and if the number of positions not matching the reference was below ⌈signature gene length * (0.0001/median depth)⌉, the reference strain was assigned. Otherwise, minor alleles with depth ≤1% of total depth were designated as noise, and the median depth of non-noise sites was used for quantification.

Strain calls that had two detected strains with estimated depths beyond 33% of the median depth or with a coefficient of variation of depths > 1 were rejected, re-analyzed under the “unknown baseline” scenario (if not already done so), and ultimately assigned as undetermined.

### 2.15 Metabolomics

Quantified metabolite data from the targeted panels were processed using normalized peak areas or absolute concentrations. Metabolites detected in at least 10% of samples and in a minimum of five samples per treatment group were retained for analysis. Peak areas were power-transformed to stabilize variance and reduce the influence of outliers. Linear regression models were fitted for each metabolite using the formula:


Metabolite feature∼Treatment+(1∣Study Site)

Features below the limit of detection (LOD) were retained as numerical values representing analytical noise. Multiple testing correction was performed using the Benjamini–Hochberg method to control the false discovery rate (FDR < 0.10).

Metabolites were grouped into KEGG and non-KEGG clusters. KEGG clusters were generated using reference pathways containing at least two compounds with ≥ 10% coverage, while non-KEGG clusters included predefined biochemical groups such as total, branched-, and unbranched-chain SCFAs, and the tricarboxylic acid (TCA) cycle metabolites. Cluster values were aggregated, LOD values imputed as 0.5 × LOD, and data log2-transformed prior to analysis. Boxplots were generated to visualize distribution patterns and effect sizes across timepoints.

### 2.16 Cytokine panels

Cytokine concentrations were interpolated from 4-parameter logistic calibration curves (1/Y^2^ weighting) generated using MSD Discovery Workbench software. Results were reported in pg./mL, with values outside the quantification range designated as below (BLQ) or above (ALQ) the limit of quantification.

## 3 Results

### 3.1 Participant disposition and baseline characteristics

Between July 3 and October 30, 2023, 46 infants were screened at five sites in Pakistan with six infants that did not meet eligibility or withdrew before randomization (Figure 1B). Forty infants were randomized (20 per group) (Figure 1B). Enrollment was terminated early due to operational challenges that led to early study closure (see Methods). Three participants discontinued dosing during the 28-day period (two Bi-26, one placebo) but remained in follow-up. Thirty-seven infants completed the study; one infant in each group died before d56 (Figure 1B). One placebo participant was lost to follow-up between d56 and d90.

Baseline characteristics were generally comparable between groups (Table 1). At d1, the mean age was 62 days, and most infants had WAZ ≤ −3 (60% [12/20] Bi-26, 70% [14/20] placebo). Half of the participants were female, and all were Asian, predominantly Punjabi. Recent antibiotic use (<30 days before enrolment) occurred in 25% (5/20) of infants in each group. From birth to screening, exclusive breastfeeding feeding was reported in 60% (12/20) of Bi-26 and 45% (9/20) of placebo participants, with the remainder mixed-fed. No HIV exposure or infection was reported. Birth by caesarean section was reported in 50% (10/20) of infants in Bi-26 and 35% (7/20) in placebo groups (Table 1). Most households used treated water sources by either boiling or using a filter (70% [14/20] Bi-26, 65% [13/20] placebo) (Table 1).

**Table 1:** Key demographics and baseline characteristics.

CONSTELLATION Groups	Bi-26 (*N* = 20)	Placebo (*N* = 20)	Total (*N* = 40)
Age (days)			
Mean (SD)	57.6 (21.1)	66.6 (23.6)	62.1 (22.6)
Median	50.0	60.0	57.5
Min, Max	31.0, 97.0	38.0, 117.0	31.0, 117.0
Age group, *n* (%)			
30–59 days	11 (55.0)	10 (50.0)	21 (52.5)
60–120 days	9 (45.0)	10 (50.0)	19 (47.5)
Sex, *n* (%)			
Female	10 (50.0)%	10 (50.0)%	20 (50.0)
Male	10 (50.0)%	10 (50.0)%	20 (50.0)
WAZ			
Mean (SD)	−3.39 (1.03)	−3.62 (0.97)	−3.50 (0.99)
Median	−3.20	−3.59	−3.37
Min, Max	−5.79, −2.21	−5.15, −2.22	−5.79, −2.21
WAZ category, *n* (%)			
>−3	8 (40.0)	6 (30.0)	14 (35.0)
≤−3	12 (60.0)	14 (70.0)	26 (65.0)
Birth weight (grams)^1^			
Mean (SD)	1907 (586.3)	2,145 (767.3)	2012 (667.9)
Median	1900	2000	2000
Min, Max	1,000, 2,800	1,200, 3,500	1,000, 3,500
Gestational age (weeks)^2^			
Mean (SD)	34.4 (2.4)	35.5 (2.9)	34.9 (2.7)
Median	34.0	37.0	35.5
Min, Max	30.0, 38.0	30.0, 39.0	30.0, 39.0
Birth delivery method, *n* (%)			
C-section	10 (50.0)	7 (35.0)	17 (42.5)
Vaginal birth	10 (50.0)	13 (65.0)	23 (57.5)
Primary feeding type since birth, *n* (%)			
Exclusively breastmilk	12 (60.0)	9 (45.0)	21 (52.5)
Mixed feeding	8 (40.0)	11 (55.0)	19 (47.5)
Antibiotics within 30 days prior to 1st dose, *n* (%)			
Yes	5 (25.0)	5 (25.0)	10 (25.0)
No	15 (75.0)	15 (75.0)	30 (75.0)
Home water source, *n* (%)^3^			
Treated water	14 (70.0)	13 (65.0)	27 (67.5)
Untreated water	6 (30.0)	7 (35.0)	13 (32.5)

### 3.2 Consistent adherence supported effective *B. infantis* engraftment

**Figure 2:**
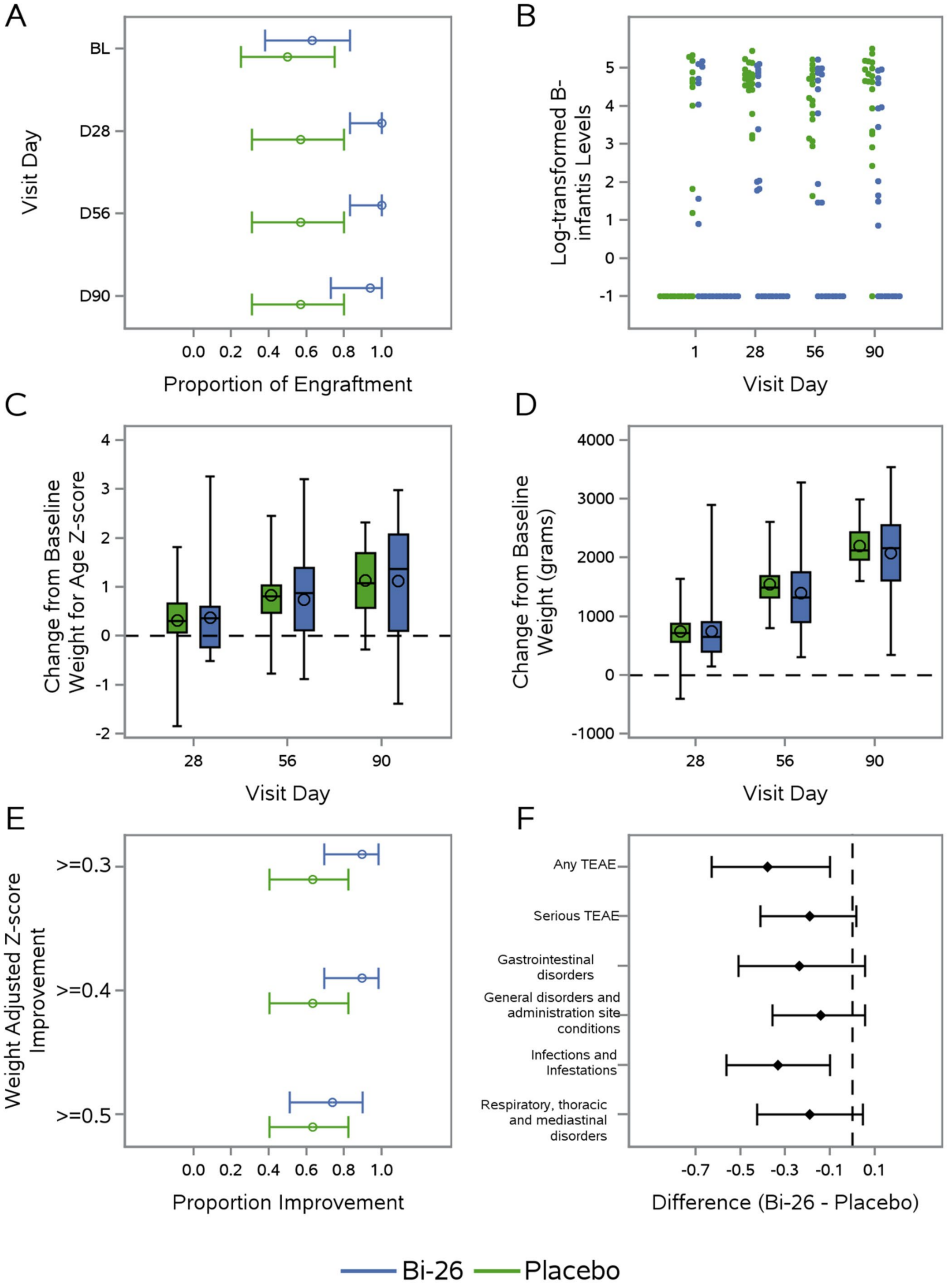
CONSTELLATION trial outcomes: Engraftment, growth, and safety. Across all panels, the Bi-26 group is shown in blue and the placebo group in green. Study visits include d1 (baseline, BL, pre-supplementation), d28 (end of supplementation), d56 (approximately 30 days post-supplementation and the primary endpoint), and d90 (final follow-up visit). **(A)** Proportion of infants with detectable *B. infantis* (>LLOQ) at all visits. **(B)** Log-transformed fecal *B. infantis* levels (genome/ng) measured across study visits. **(C)** Change from baseline in weight-for-age *Z*-score (WAZ) at d28, d56, and d90.4. **(D)** Change from baseline in weight (grams) at d28, d56, and d90. **(E)** Proportion of infants achieving predefined thresholds of WAZ improvement (>0.3, >0, >0.5) at d56. **(F)** Between-group differences in the incidence of adverse events through d90 by system organ class for which there was at least one event in the Bi-26 group.

Full compliance (receipt of all 28 doses) was achieved in 75% (15/20) of participants in the Bi-26 group and 55% (11/20) in the placebo group. At least 50% of total feedings from breastmilk were recorded by the caregiver through d56 in 90% (18/20) Bi26 group and 85% (17/20) placebo group (data not shown). Total *B. infantis levels* were measured in infant stool using a validated qPCR assay (13). At d1, *B. infantis* was detected in half of the infants in each group (Figure 2A). By d28 and 56, all infants in Bi26 group had detectable *B. infantis* levels compared to approximately half in the placebo group. Median levels of *B. infantis* in fecal samples (log10 genome/ng) were higher in the Bi-26 group at d28 (4.73, Bi-26 vs. 1.80, placebo; *p* = 0.001), d56 (4.58, Bi-26 vs. 1.47, placebo; *p* = 0.035), and d90 (4.67, Bi-26 vs. 1.57, placebo; *p* = 0.001) (Figure 2B). We found no significant differences between changes in fecal pH from d1 to d28 between groups (data not shown).

### 3.3 No significant effect of Bi-26 supplementation on growth outcomes

There was no significant difference between groups in the change in WAZ (0.78, Bi-26 vs. 0.67, placebo [*p* = 0.7]) (Figure 2C) or change in weight in grams (1491.5, Bi-26 vs. 1382.2, placebo [*p* = 0.6]) from d1 to d56 (Figure 2D). Improvements of WAZ of at least 0.3 at d56 were observed in 87.5% (14/16) of infants in the Bi-26 group and 64.3% (9/14) in the placebo group (PP population) (Figure 2E). The proportion of infants no longer considered underweight (WAZ score > − 2) at d56 was similar between groups (data not shown). No participants in the Bi-26 group and one in the placebo group was re-hospitalized for an acute non-surgical illness through the d56 visit.

### 3.4 Fewer adverse events in infants receiving Bi-26 supplementation

Adverse events (AEs) were less frequent in the Bi-26 group (8/20 participants [40%], 17 events) than in the placebo group (16/20 [80%], 49 events) (Figure 2F, Table 2). Serious adverse events (SAEs) occurred in one Bi-26 participant (one event) and five placebo participants (nine events) (Figure 2F, Table 2). Within the infections and infestations system organ class, only one event occurred in one Bi-26 participant compared with 14 events in eight placebo participants (Figure 2F, Table 2). In the Bi-26 group, abdominal pain and vomiting were the most common AEs reported by 4 participants each; in the placebo group, vomiting (8 participants), and abdominal pain, diarrhea, pyrexia, and cough (3 participants each) predominated (Table 2). Most events were mild. One SAE (pyrexia) deemed related to study intervention by the investigator occurred in the placebo group. Independent of supplementation group, 77% of infants from untreated home water sources (10/13) experienced AEs compared to 52% infants from households with treated water sources (14/27) (data not shown). There was one participant death in the Bi-26 group (preferred term of ‘death’) and 1 participant death in the Placebo group (preferred term of ‘sudden death’). Both deaths occurred after the study intervention period had completed but prior to the d56 primary analysis endpoint and were not considered related to the study intervention by both investigator and sponsor.

**Table 2:** Overall summary of adverse events (AEs) (safety population).

System organ class (SOC)/Preferred term (PT)	Bi-26 (*N* = 20) *n* (%) E	Placebo (*N* = 20) *n* (%) E
Any AE	8(40.0) 17	16 (80.0) 49
Any TEAE	8 (40.0) 17	16 (80.0) 49
Study intervention related TEAE	0	1 (5.0) 1
Maximum severity of TEAE		
Mild	6 (30.0) 13	9 (45.0) 36
Moderate	1 (5.0) 3	2 (10.0) 5
Severe	1 (5.0) 1	5 (25.0) 8
TEAE leading to intervention discontinuation	0	1 (5.0) 1
TEAE leading to withdrawal	1 (5.0) 1	1 (5.0) 1
Serious TEAE	1 (5.0) 1	5 (25.0) 9
Serious TEAE with outcome of death	1 (5.0) 1	1 (5.0) 1
TEAEs reported in ≥ 1 participant in any group		
Congenital, familial and genetic disorders	0	1 (5.0) 1
Hydrocele	0	1 (5.0) 1
Gastrointestinal disorders	6 (30.0) 13	11 (55.0) 21
Abdominal pain	4 (20.0) 6	3 (15.0) 6
Vomiting	4 (20.0) 5	8 (40.0) 10
Abdominal distension	1 (5.0) 5	0
Diarrhea	1 (5.0) 5	3 (15.0) 4
Gastroesophageal reflux disease	0	1 (5.0) 1
General disorders and administration site conditions	1 (5.0) 1	4 (20.0) 6
Death	1 (5.0) 1	0
Pyrexia	0	3 (15.0) 5
Sudden death	0	1 (5.0) 1
Infections and infestations	1 (5.0) 1	8 (40.0) 14
Nasopharyngitis	1 (5.0) 1	2 (10.0) 2
Acarodermatitis	0	1 (5.0) 1
Bronchiolitis	0	1 (5.0) 1
Conjunctivitis viral	0	1 (5.0) 1
Influenza	0	1 (5.0) 1
Measles	0	1 (5.0) 1
Meningitis	0	1 (5.0) 1
Otitis media	0	1 (5.0) 1
Pneumonia	0	2 (10.0) 3
Pneumonia measles	0	1 (5.0) 1
Upper respiratory tract infection	0	1 (5.0) 1
Respiratory, thoracic and mediastinal disorders	2 (10.0) 2	6 (30.0) 6
Bronchial hyperreactivity	1 (5.0) 1	0
Oropharyngeal discomfort	1 (5.0) 1	1 (5.0) 1
Cough	0	3 (15.0) 3
Nasal congestion	0	1 (5.0) 1
Respiratory distress	0	1 (5.0) 1
Skin and subcutaneous tissue disorders	0	1 (5.0) 1
Seborrheic dermatitis	0	1 (5.0) 1

### 3.5 Bi-26 supplementation was associated with temporal shifts in microbiome diversity, composition, and function

**Figure 3:**
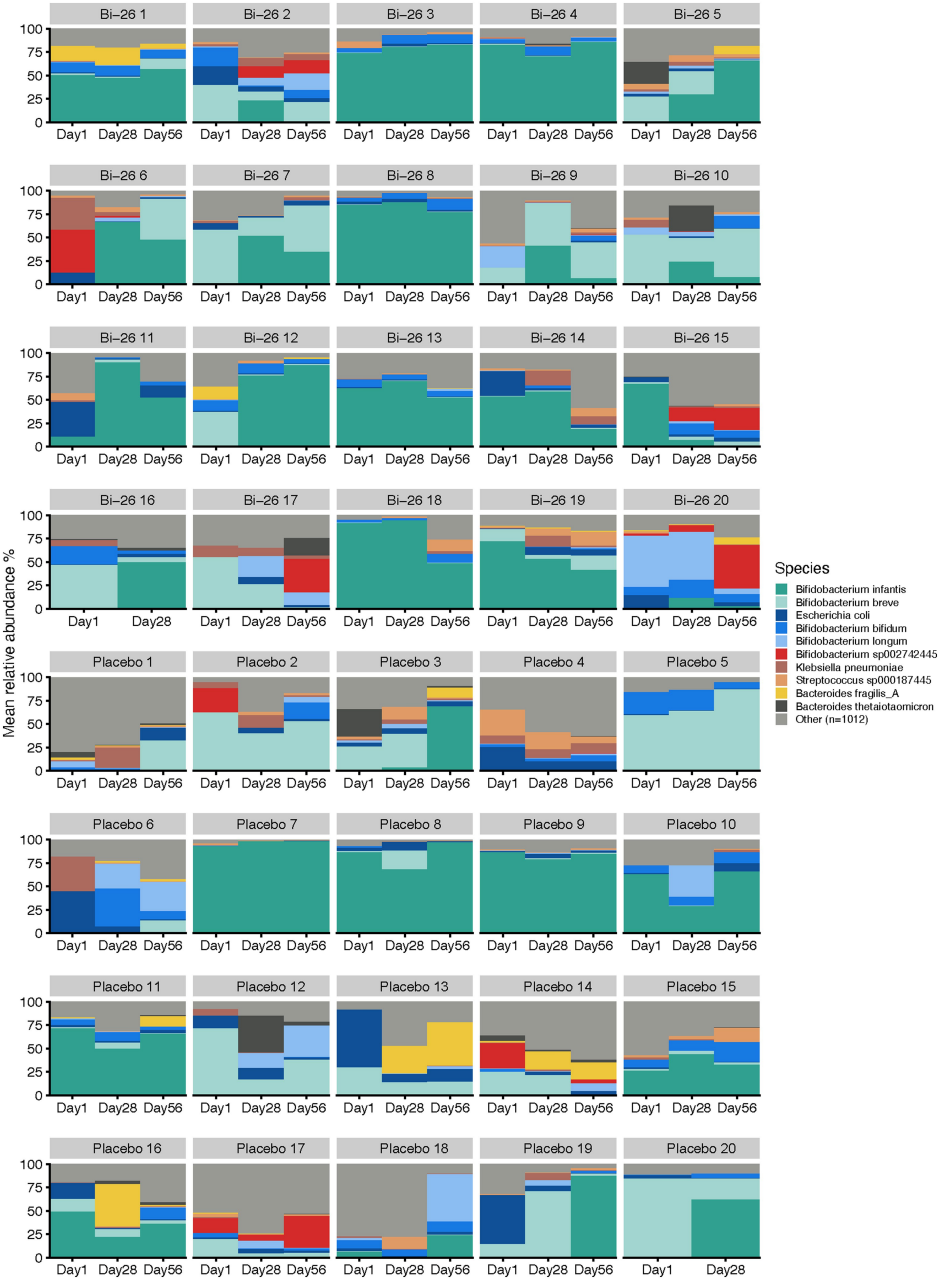
Species-level taxonomic overview stratified by group and participant. For each timepoint, the bar plot indicates the relative abundance of the 10 species with highest average abundance across all samples. Light grey (Other) indicates the total relative abundance of all other species (*n* = 1,012).

We assessed the broader effects of Bi-26 supplementation on gut microbiome composition using shotgun metagenomic sequencing. Taxonomic profiles from both groups highlighted strong individuality in microbial community structure, with some infants maintaining relatively stable compositions throughout the study period, while others exhibited notable fluctuations (Figure 3). *Bifidobacterium* remained one of the most abundant genera across all subjects, and most infants were colonized with either *B. infantis* or *B. breve* over time (Figure 3).

Alpha diversity was measured with species richness and Shannon diversity from rarefied species abundances. While there were no significant differences between the groups in species richness at any given timepoint (Supplementary Figure 1A), the Shannon diversity was lower in the Bi-26 group at d28 compared to placebo (*p* = 0.04) (Supplementary Figure 1B). Beta diversity based on Bray–Curtis dissimilarities revealed that taxonomic composition was similar between groups at d1 but diverged by d28 following supplementation, with this distinction diminishing by d56 (Supplementary Figure 2). Samples tended to cluster by study site, reflecting local effects, but Bi-26 microbiomes were more tightly clustered at d28, suggesting greater compositional uniformity after supplementation (Supplementary Figure 2). A permutational multivariate analysis of variance (PERMANOVA) confirmed a significant difference in Bray–Curtis distances between groups at d28, with treatment explaining 8.4% of variance in microbial composition; no significant differences were observed at d1 or 56 (Supplementary Table 1).

**Figure 4:**
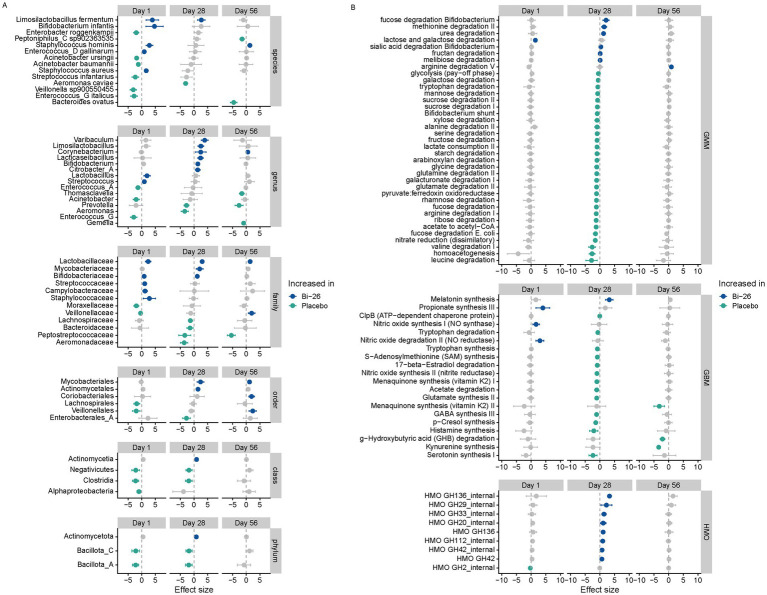
Taxonomic and functional differences in the gut microbiome between Bi-26 and placebo groups. **(A)** Forest plots showing effect sizes for microbial taxa comparing placebo to Bi-26 across study timepoints. Positive effect sizes (right of the dashed vertical line) indicate higher relative abundance in the Bi-26 group. Points represent estimated effect sizes, bars show 95% confidence intervals. Colored points (blue for Bi-26, green for placebo) denote taxa with false discovery rate (FDR) < 0.1; gray points indicate FDR ≥ 0.1. Taxa without displayed intervals did not meet prevalence criteria for modeling. **(B)** Forest plots showing effect sizes for microbial functional modules comparing placebo to Bi-26 across study timepoints. Positive effect sizes (right of the dashed vertical line) indicate higher abundance in the Bi-26 group. Points represent estimated effect sizes, bars show 95% confidence intervals. Colored points indicate FDR < 0.1 and gray points indicate FDR ≥ 0.1.

We further interrogated differences between Bi-26 and placebo groups in taxonomical composition and in functional gene abundance. D1 taxonomic composition differences were observed between Bi-26 and placebo groups at all levels, which shifted following supplementation at d28 and again at d56 (Figure 4A). In contrast, functional gene profiles were highly similar between groups at d1 but showed significant divergence at d28, which largely reverted to baseline by d56 (Figure 4B). Notably, *B. infantis* abundance in this analysis was significantly higher in the Bi-26 group at d1 but not at d28 (*p* = 0.1) or d56 (*p* = 0.8) (Figure 4A). By d28, however, both the genus *Bifidobacterium* and the family Bifidobacteriaceae were elevated in the Bi-26 group relative to placebo (Figure 4A). These taxonomic differences coincided with increased fucose and sialic acid degradation pathways and elevated levels of several HMO-specific glycosyl hydrolases in the Bi-26 group at d28 (Figure 4B).

### 3.6 Baseline *B. infantis* composition shapes Bi-26 engraftment and persistence

**Figure 5:**
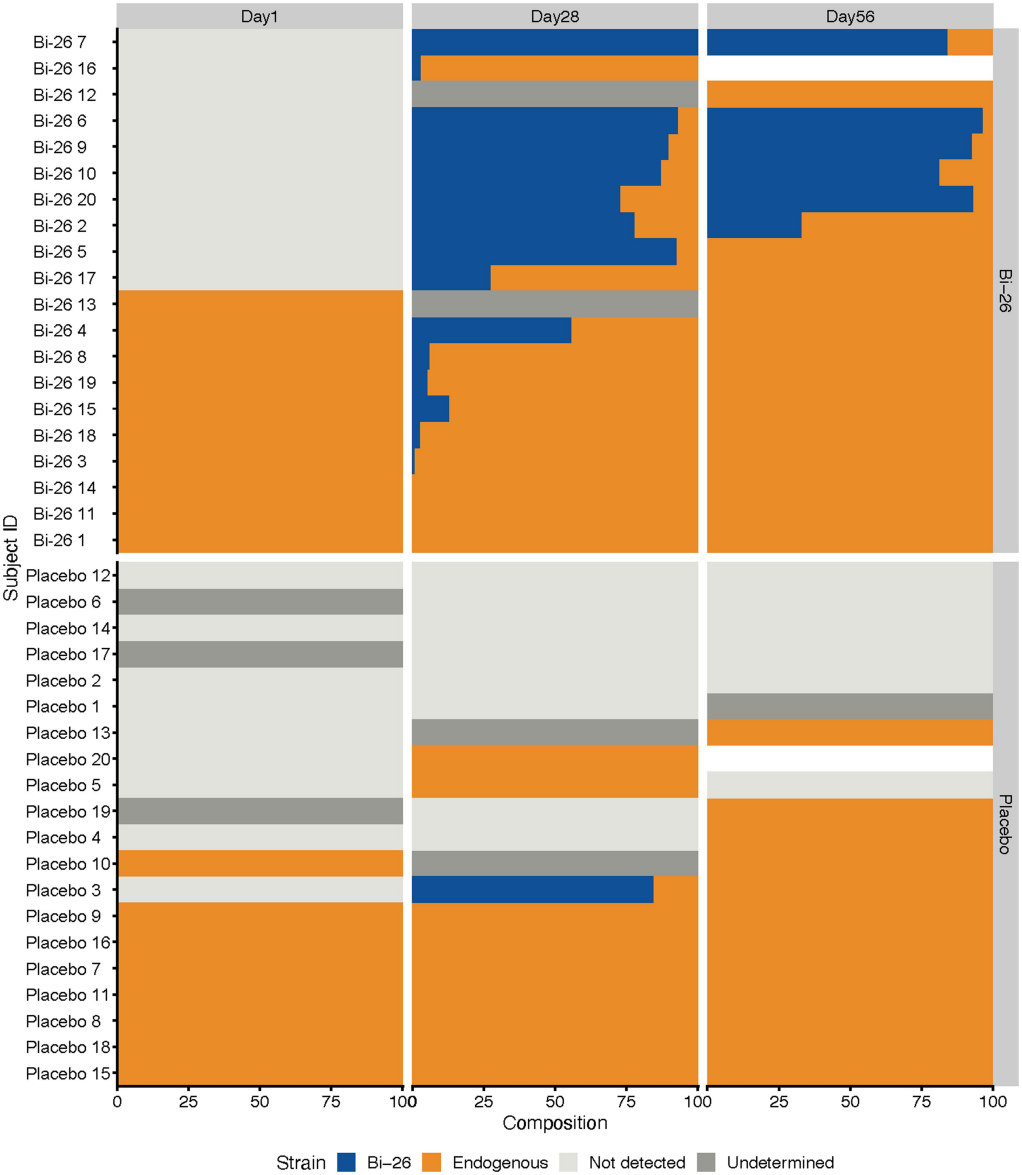
*Bifidobacterium infantis* strain-level composition in Bi-26 vs. placebo groups across study visits. Each bar represents the proportion of *B. infantis* strain types detected within a sample for each participant at d1, d28, and d56. Panels show infants in the Bi-26 group (top) and placebo group (bottom). Percent composition reflects the relative contribution of endogenous *B. infantis* strains versus the Bi-26 strain among all *B. infantis* reads detected. Not detected (light gray): *B. infantis* was not detected in the sample. Undetermined (gray): Strain type could not be reliably assigned due to low signature-gene coverage or high noise. Endogenous (orange): *B. infantis* strains not matching the Bi-26 profile. Bi-26 (blue): Reads assigned to the supplemented Bi-26 strain. White bars indicate missing samples.

A single-nucleotide-variant (SNV)–based approach was used to differentiate between endogenous *B. infantis* and the administered Bi-26 strain. Endogenous *B. infantis* was detected in samples across both intervention groups, with none of the d1 samples containing the Bi-26 strain (Figure 5). Among infants in the Bi-26 group, the administered strain was detected in 15 of 20 samples at d28, representing 0.6–100% of total *B. infantis* abundance, and in 6 of 20 samples at d56, comprising 32.7–96.2% of the total *B. infantis* abundance (Figure 5). Bi-26 engrafted at a high relative abundance in infants who lacked an endogenous *B. infantis* strain at baseline and persisted through d56 in those who exhibited high Bi-26 abundance on d28 (Figure 5). By d56, endogenous strains generally re-emerged as dominant, and Bi-26 levels declined following the cessation of supplementation (Figure 5). Notably, one placebo participant showed a Bi-26 match at d28, suggesting exposure to Bi-26 (Figure 5). Further investigation revealed that this participant was part of a twin pair enrolled in the study with their twin randomized to the Bi-26 group, explaining the observed Bi-26 exposure and transient colonization.

### 3.7 Bi-26 supplementation promotes transitions toward *B. infantis*–dominated community types

**Figure 6:**
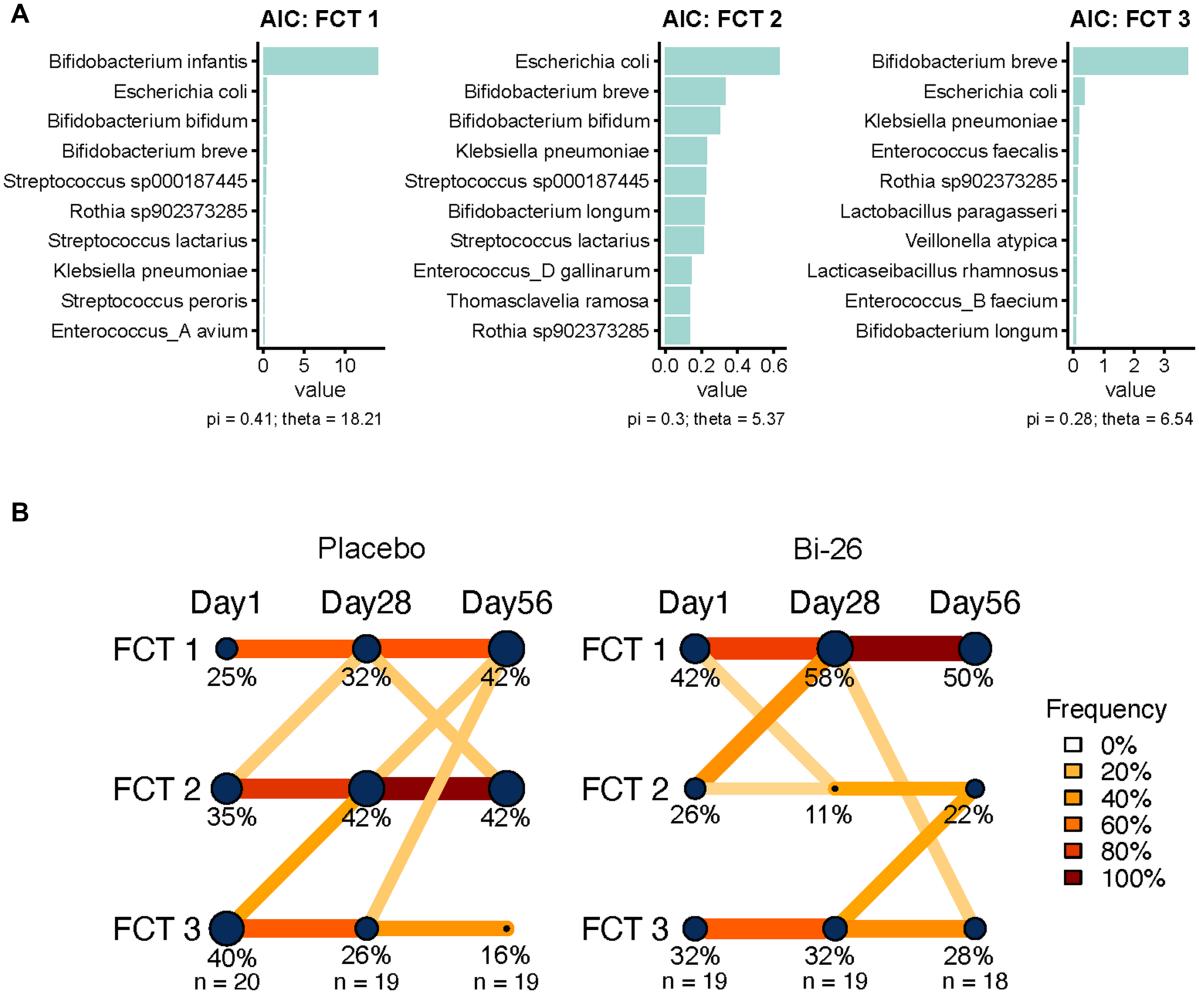
Defining microbial Fecal Community Types (FCT) and visualizing infant transitions across FCTs. **(A)** Bar plots display the top species driving each FCT, with values indicating their relative importance within the cluster. Species names reflect taxonomic annotations. Pi values represent the proportion of samples belonging to each FCT, and theta values reflect the dispersion (variability) of samples within the cluster. **(B)** Network-style diagrams illustrate how infants move between FCTs from d1 to d28 to d56. Left (placebo) and right (Bi-26). Nodes represent each FCT at each timepoint, with node size proportional to the fraction of infants assigned to that FCT (fraction of infants within a FCT are also labelled). Connecting lines indicate transitions between FCTs; line color intensity reflects the proportion of infants transitioning from one FCT to another, with values rounded up to the nearest percentage bin for visualization. Percentages within nodes indicate the distribution of infants in each FCT over time for the Bi-26 and placebo groups.

To explore whether gut microbiota composition influenced response to the intervention, we applied Dirichlet multinomial modeling (DMM) (17) to define fecal community types (FCTs). This data-driven approach identified three clusters of microbial taxa that represent distinct community structures: FCT1, FCT2, and FCT3. FCT1 was characterized by the dominance of *B. infantis*; FCT2 represented a more diverse community including *E. coli*, *B. breve*, and *B. bifidum*; and FCT3 was dominated by *B. breve* (Figure 6A). Each sample was assigned to an FCT based on its posterior probabilities obtained from the DMM modeling, enabling longitudinal tracking of FCT dynamics throughout the study. We then evaluated the stability of FCT classification over time and assessed transitions between FCTs within each study group (Figure 6B). Many infants remained within their baseline FCT, as indicated by the prominent horizontal transition lines for FCT2 in the placebo group and for FCT1 and FCT3 in both groups. In contrast, thinner non-horizontal lines reflect FCT transitions. Among infants who changed FCT over the course of the study, a greater proportion in the Bi-26 group transitioned from the more diverse FCT2 to the *B. infantis*-associated FCT1 compared with the placebo group. Interestingly, no infants in the Bi-26 group classified as the *B. breve*-associated FCT3 at baseline converted to FCT1 during the study (Figure 6B). By study end, we observed a higher proportion of infants in the placebo group were in FCT2 (42% [8/19]) compared to the Bi-26 group (22% [4/18]) (Figure 6B).

### 3.8 Transient alterations in fecal metabolites associated with Bi-26 supplementation

**Figure 7:**
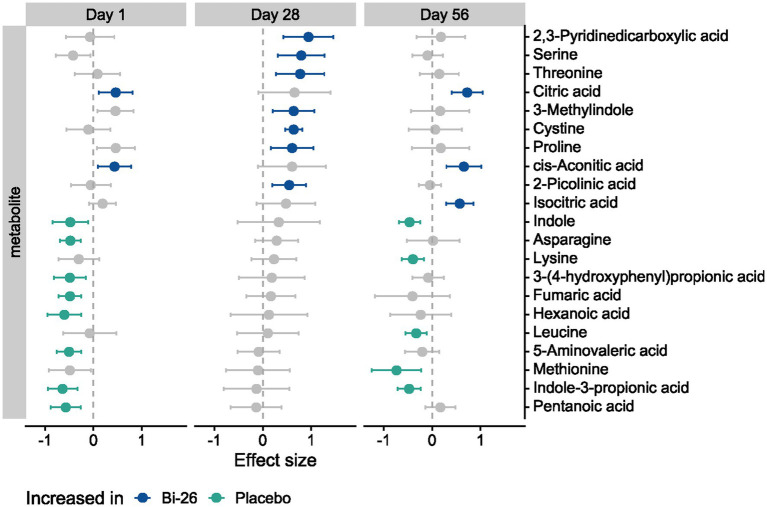
Metabolomic features associated with Bi-26 supplementation over the study period. Forest plots showing effect sizes for metabolites comparing placebo to Bi-26 at each study visit. Only features that differed significantly between groups at least one timepoint are displayed. Positive effect sizes (right of the dashed vertical line) indicate higher levels in the Bi-26 group. Points represent estimated effect sizes, and bars denote 95% confidence intervals. Colored points (blue or green) indicate features with FDR < 0.1, while gray points indicate FDR ≥ 0.1.

To evaluate whether Bi-26 affected gut metabolic activity, fecal metabolites were analyzed using targeted panels for SCFAs and tryptophan-related compounds (see Methods), pathways influenced by *B. infantis* and linked to gut integrity and immune regulation (1, 6, 10, 18). At d1, we observed significant differences between Bi-26 and placebo groups across multiple metabolites; however, on d28, none of these baseline differences persisted (Figure 7). At d28, seven metabolites were significantly elevated in the Bi-26 group compared with placebo (Figure 7). By d56, many of the metabolites that had differed at baseline again showed significant differences between groups, indicating a reversion toward baseline metabolic profiles after the cessation of supplementation (Figure 7). No significant differences in acetate or lactate levels were observed between groups at any timepoint.

### 3.9 Bi-26 supplementation does not alter fecal cytokine expression

Fecal cytokines were quantified using a validated electrochemiluminescence (ECL) assay detecting IFN*γ*, IL-1β, IL-2, IL-4, IL-6, IL-8, IL-10, IL-12p70, IL-13, and TNF to assess potential effects on mucosal immune activity. Five cytokines (IL-10, IL-12p70, IL-13, IL-4, and IL-6) were below the limit of detection in both groups. Among detectable cytokines (IFN-γ, IL-1β, IL-2, IL-8, TNF), no significant differences were observed between Bi-26 and placebo at any timepoint (Supplementary Figure 3). Although immune-related metabolites shifted at d28, the effects were not sustained, which may explain the lack of cytokine differences over time.

### 3.10 Exploratory associations of *B. infantis* abundance and Fecal community type with clinical outcomes

In a hypothesis-generating analysis, we examined whether changes in biomarker measurements were associated with clinical outcomes, including the primary endpoint of change in WAZ and the cumulative number of AEs reported through d90. Given the relatively small sample size and the limited power of this study, we restricted these analyses to a subset of biomarkers that showed durable changes by d56, the time point of the primary efficacy assessment, rather than transient fluctuations observed during the supplementation period. Accordingly, the analyses were limited to fecal *B. infantis* abundance (quantified by qPCR) and FCT assignments. Because the primary endpoint was not significant, both groups were combined to increase power for detecting associations between biomarkers and clinical outcomes.

Fecal *B. infantis* levels at d28 and d56 were not correlated with changes in WAZ at d56 (d28: *r* = 0.02, *p* = 0.89; d56 *r* = 0.04, *p* = 0.81; data not shown). However, a modest negative correlation was observed between *B. infantis* levels at d28 and the total number of AEs reported during the study (*r* = −0.315, *p* = 0.04) (Supplementary Figure 4A). No such relationship was seen at d56 (Supplementary Figure 4B), suggesting that this association was most apparent at the peak of *B. infantis* colonization following supplementation. For analysis based on FCT, we combined the two *Bifidobacterium*-dominated community types (FCT1 and FCT3) and compared them to the more compositionally diverse community type (FCT2). This grouping, based on prior evidence linking *B. infantis*/*B. breve* enrichment and healthy infant microbiomes (9, 19–21), reduced comparisons and improved interpretability of microbiome–clinical relationships. No significant association was detected between changes in WAZ from d1 to d56 and FCT at d1 (*p* = 0.8, data not shown) or d56 (*p* = 0.13, Supplementary Figure 5A). However, while FCT at d1 was not related to the total number of AEs accumulated through the study (*p* = 0.19, data not shown), infants classified as FCT2 at d56 experienced a higher number of AEs over the study period compared with those in *Bifidobacterium*-dominated FCT1/3 communities (*p* = 0.03, Supplementary Figure 5B).

## 4 Discussion

The double-blind, randomized controlled CONSTELLATION trial in underweight infants in Pakistan compared *B. infantis* Bi-26 with placebo and found that, although fecal *B. infantis* levels increased with Bi-26 supplementation, there were no meaningful between-group differences in changes in WAZ or weight (g); however, the final sample size was 10% of the intended sample size and thus limited the ability to detect statistically significant differences between groups. Notably, fewer AEs were reported in the Bi-26 group, and exploratory analyses suggest that AE frequency may be inversely associated with fecal *B. infantis* levels and/or dominance of Bifidobacterium community types in early infant life.

This study has several important limitations. Early trial discontinuation substantially reduced the sample size, limiting power to detect differences in primary and secondary endpoints and preventing robust multivariable analyses integrating clinical and biomarker measures. We also observed some d1 differences in laboratory and exploratory measures, likely reflecting baseline variation that was amplified by the smaller-than-planned sample size that may not have persisted in the fully enrolled population. However, this dataset still provides an informative platform to build upon when designing larger trials to evaluate the effects of *B. infantis* supplementation on growth and other health outcomes, and to further explore mechanistic hypotheses.

Although the CONSTELLATION trial enrolled fewer participants than planned, its sample size was comparable to that of SYNERGIE; nevertheless, CONSTELLATION did not reproduce the growth improvements reported in SYNERGIE (12). Importantly, there were some key differences between the two studies that may help explain the divergent findings. The SYNERGIE study included older infants with a high prevalence of baseline pedal edema and specifically targeted those hospitalized for severe acute malnutrition under standardized management, including nutritional rehabilitation units (12). In contrast, the CONSTELLATION trial enrolled underweight infants hospitalized for acute non-surgical illness and not specifically for severe acute malnutrition. Furthermore, Barratt et al. reported lower or absent *B. infantis* in Bangladeshi infants with severe acute malnutrition versus healthy controls (12). While the CONSTELLATION trial did not have a healthy comparator arm, over half of underweight Pakistani infants enrolled carried detectable or high *B. infantis* levels, suggesting possible geographic variation in *B. infantis* abundance among malnourished infants. The trials also differed in endpoints and analytic approaches, which may have influenced outcome comparability.

CONSTELLATION’s exploratory biomarker analyses suggest biological reasons why a weight effect may not have been detectable, specifically that our hypothesized mechanistic pathway may have been initiated but was not sustained in study participants. We hypothesized that *B. infantis* supplementation would promote a *B. infantis*–dominant microbiome that lowers intestinal pH, curbs pathogen overgrowth, and generates metabolites that support barrier repair and balanced immunity (9), thereby improving nutrient absorption and growth. At d56 in the CONSTELLATION trial, 4 weeks after the study intervention period had completed, Bi-26 supplementation increased total *B. infantis* levels (by qPCR) and shifted community structure (by FCT analysis); however, we observed no between-group differences in fecal pH, notable reductions of potentially pathogenic taxa, acetate or lactate levels, or fecal cytokine shifts. Many shifts in functional gene abundance and downstream metabolic outputs were transient and confined to the supplementation period. The absence of durable functional remodeling across the mechanistic domains of our hypothesis may help account for the limited effects of Bi-26 supplementation on WAZ and weight in this small cohort. The observation that functional markers returned toward baseline by Day 56 opens the possibility that longer probiotic dosing durations (>28 days), continued supplementation, or alternative dosing regimens could be explored to better understand how to sustain functional remodeling in the infant gut. Future trials may benefit from systematically evaluating different dosing durations and frequencies to assess whether more durable functional effects can be achieved and whether such effects translate into improvements in growth.

Furthermore, strain-tracking analysis demonstrated that Bi-26 engraftment occurred almost exclusively in infants who lacked endogenous *B. infantis* at baseline. In these infants, supplementation appeared to facilitate the subsequent growth of endogenous *B. infantis* lineages by d56, suggesting that probiotic introduction can transiently reshape the gut ecosystem to better support colonization by native strains. Notably, some of the infants without endogenous *B. infantis* at baseline belonged to the FCT3 community type, which was characterized by dominance of *B. breve*. Although both probiotic and endogenous *B. infantis* strains expanded in these infants, transitions from FCT3 to FCT1, the *B. infantis*–dominated community type, were not observed in the Bi-26 group during the study period. This finding is consistent with prior evidence that early colonization by *B. breve* can occupy overlapping ecological niches, making subsequent establishment of *B. infantis* more difficult (22, 23). Together, these results underscore the importance of ecological niche availability as a determinant of probiotic engraftment and suggest that baseline microbiome composition may shape mechanistic responses to probiotic supplementation.

A key methodological finding was that *B. infantis* abundance estimates differed by the measurement approach, highlighting important distinctions between qPCR-based quantification and metagenomic profiling. The qPCR assay provided direct quantification of *B. infantis* genome equivalents and showed significant increases in *B. infantis* levels in the Bi-26 group at d28 and d56. In contrast, shotgun metagenomics, which measures relative abundance within the total microbial community, showed higher *B. infantis* levels in the Bi-26 group at d1 and no between-group differences over time. Because metagenomic data are compositional (24, 25), relative abundance may appear unchanged despite true increases in absolute load, particularly when other taxa expand or total biomass varies. Thus, while both methods assess *B. infantis*, they capture different dimensions of colonization. In this study, engraftment was defined as a secondary endpoint, and the success of engraftment was pre-defined to be evaluated using a validated qPCR method for quantification of *B. infantis* levels. Exploratory analyses were then conducted to characterize broader microbiome shifts using metagenomic data. Future studies should prespecify engraftment definitions and consider use of targeted or metagenomic assays based on trial objectives.

Fewer AEs were reported among infants receiving Bi-26 compared to placebo. However, interpretation of this finding is constrained given the small sample size and potential cofounding factors and as such, it is not possible to determine whether this difference reflects a true supplementation effect or a chance finding. Notably, the SYNERGIE study in malnourished infants reported no group differences in AEs (12), and recent trials in healthy infants conducted in both high-income (26, 27) and LMIC settings (28) similarly found *B. infantis* supplementation (alone or in combination with other probiotic strains) to have acceptable safety profile but without measurable effects on AE frequency. Future studies should incorporate larger sample sizes and standardized AE monitoring to rigorously evaluate the potential for *B. infantis* to reduce illness burden in early life. In addition, they should assess the influence of exclusive breastfeeding, sanitation, and hygiene practices on infant growth, and determine whether *B. infantis* supplementation can further enhance outcomes within these contexts. Interestingly, our exploratory analyses identified correlations between higher fecal *B. infantis* abundance as measured by qPCR and fewer reported AEs, as well as lower AE frequency in participants with *Bifidobacterium*-dominated community types, raising the possibility that these measures could serve as candidate biomarkers of reduced illness burden in early infant life. In the absence of comparable AE datasets from prior trials, this study provides a foundational resource that can inform future hypothesis-driven investigations and support the design of studies evaluating associations between microbiome-derived biomarkers and health outcomes.

Although the hypothesized impact on weight gain was not observed in this small trial, the findings support the feasibility and safety of *B. infantis* supplementation and provide a rationale for larger, adequately powered studies to assess biological effects and reproducibility across infant populations. Future studies should build on this framework to test how baseline *B. infantis* status, dosing duration, and breastfeeding practices influence long-term functional remodeling, and whether sustained metabolic and functional changes are required to achieve measurable clinical benefits. Given the substantial morbidity and mortality among underweight infants in LMICs, identifying interventions that correct gut microbial imbalances and translate into improved growth or reduced illness burden could have a meaningful global impact on infant health.

## Data Availability

The datasets presented in this article are not readily available because the data is protected by clinical trial privacy regulations and informed consent agreements. Anonymized participant level data may be shared upon request, in accordance with the trial participants' written and executed informed consent document and any local or applicable regulations on data sharing. Researchers may submit a request for anonymized participant level data, along with a research proposal, to corresponding author.
