# Supplementary material for: Impact of Bifidobacterium infantis supplementation on growth, health outcomes, and gut microbiome features in underweight infants from Pakistan
Source: Front Nutr. 2026 Apr 29;13:1783141. doi: 10.3389/fnut.2026.1783141 (PMC13167951; doi:10.3389/fnut.2026.1783141)
Supplement: Supplementary file 1 [file Supplementary_file_1.docx]

Supplementary Material

**Supplement to: Parrino J, Sunshine J, Tripp K et al. Impact of *Bifidobacterium infantis* supplementation on growth, health outcomes, and gut microbiome features in underweight infants from Pakistan**

| Supplementary Figure 1: Alpha Diversity. Boxplots showing differences in (A) species richness and (B) Shannon diversity index per in Bi-26 (blue) vs. placebo (green) groups stratified by visit. Statistics were assessed using a linear mixed model with the intervention as a fixed effect and the study site as a random effect. P-values are shown only for the significant comparisons (p < 0.05). |
| --- |
| **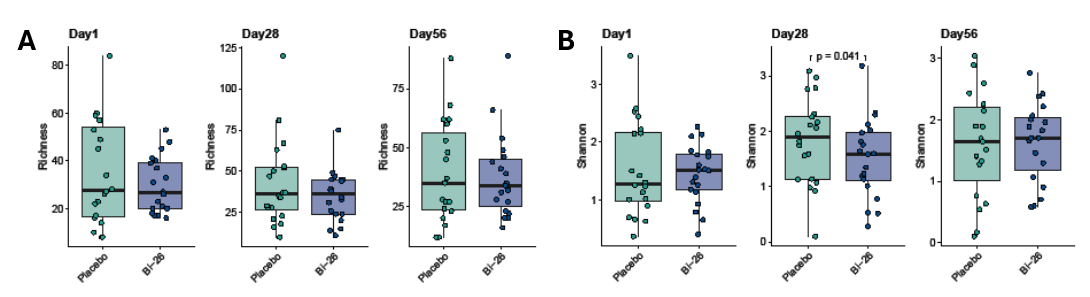** |

| Supplementary Figure 2: Beta Diversity. Principal coordinates analysis based on Bray–Curtis dissimilarities among samples, calculated based on the species abundances. Samples are color-coded (Bi26, blue; placebo, green) with shapes representing different study sites. The x- and y-axis labels indicate the microbial variance explained by the first two principal coordinates. |
| --- |
| **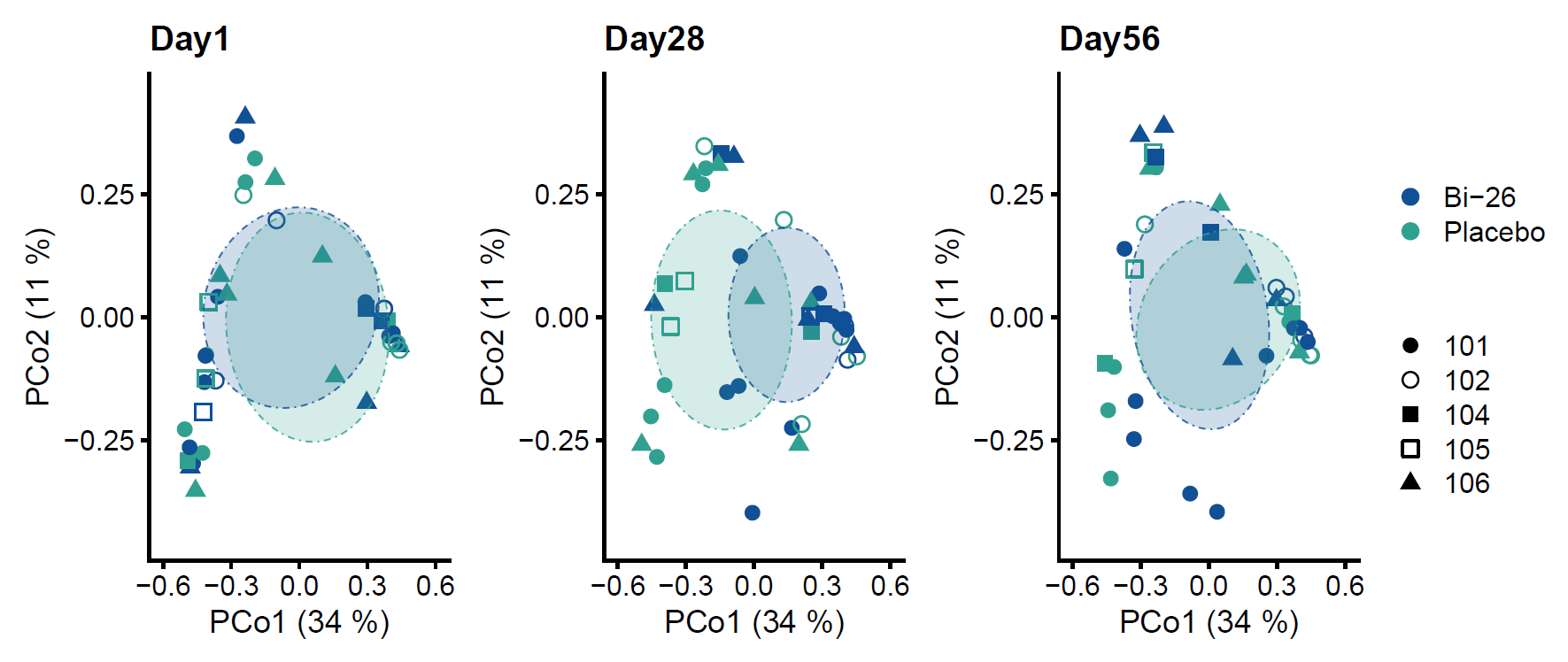** |

| Supplementary Table 1: PERMANOVA tests on Bray-Curtis distances between Bi-26 and placebo groups. The R^2^ can be interpreted as variance explained by the variable(s). | | | |
| --- | --- | --- | --- |
| **Variable(s)** | **R^2^** | **p-value** | **Timepoint** |
| Intervention + site | 1.77% | 0.595 | Day 1 |
| Intervention | 1.43% | 0.773 | Day 1 |
| Intervention + site | 9.76% | 0.002 | Day 28 |
| Intervention | 8.43% | 0.007 | Day 28 |
| Intervention + site | 2.32% | 0.389 | Day 56 |
| Intervention | 2.65% | 0.419 | Day 56 |

| Supplementary Figure 3: Quantification of cytokines in stool. Fecal cytokines were quantified using a validated electrochemiluminescence (ECL) assay. Cytokine levels above LLOQ are displayed below in Bi-26 (blue) and placebo (green) groups at d1, d28, and d56. Five cytokines (IL-10, IL-12p70, IL-13, IL-4, and IL-6) were below the limit of detection in both groups. |
| --- |
| **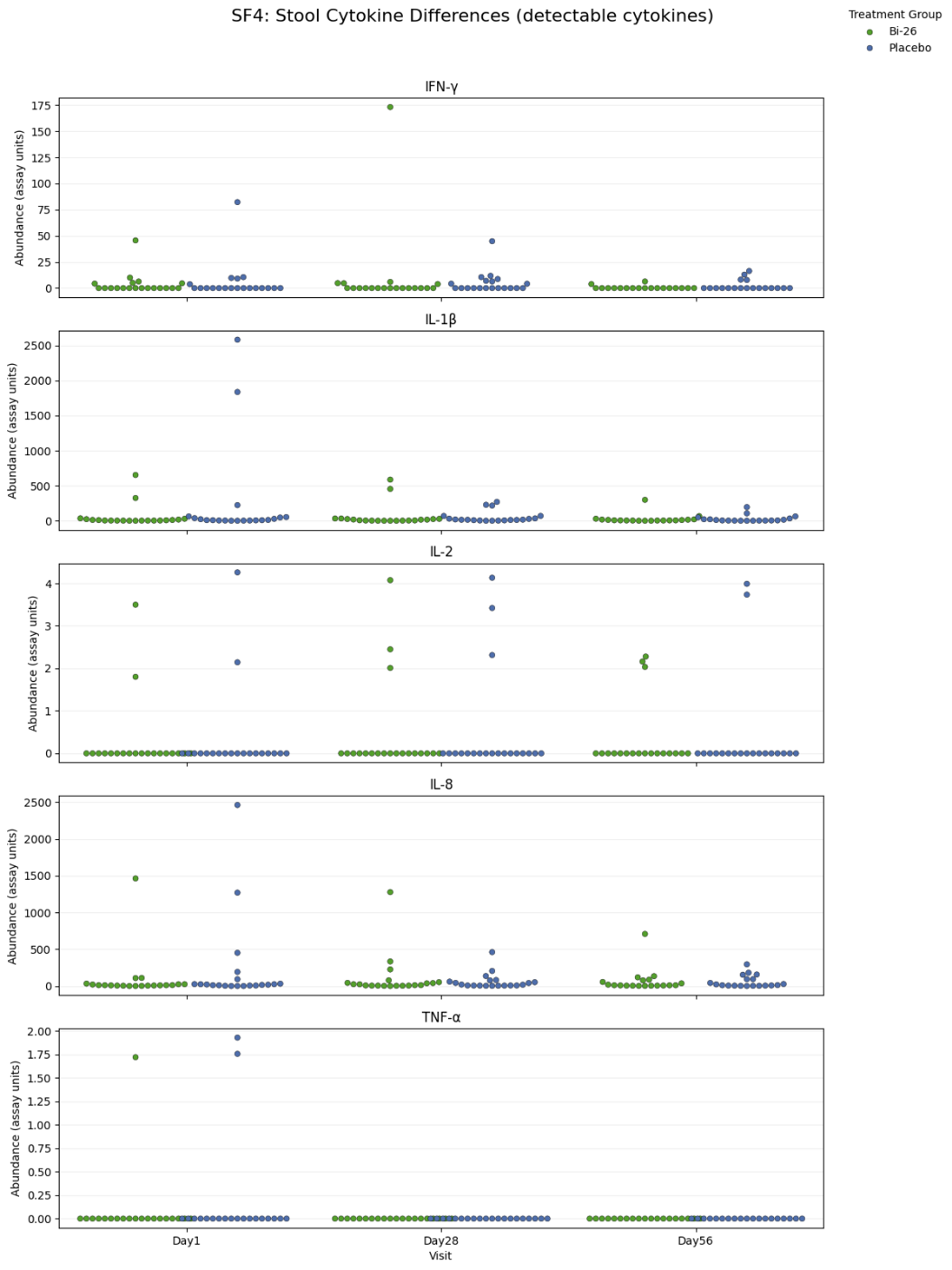** |

| Supplementary Figure 4: Correlations between fecal B. infantis levels and CONSTELLATION outcomes. Correlations shown for fecal *B. infantis* levels (by qPCR, log-transformed genomes/ng) and cumulative number of adverse events reported through day 90. (A) d28 fecal *B. infantis* levels (r= –0.315, p = 0.04). (B) d56 fecal *B. infantis* levels (r= –0.129, p = 0.44). Bi-26 (blue), placebo (green) |
| --- |
| **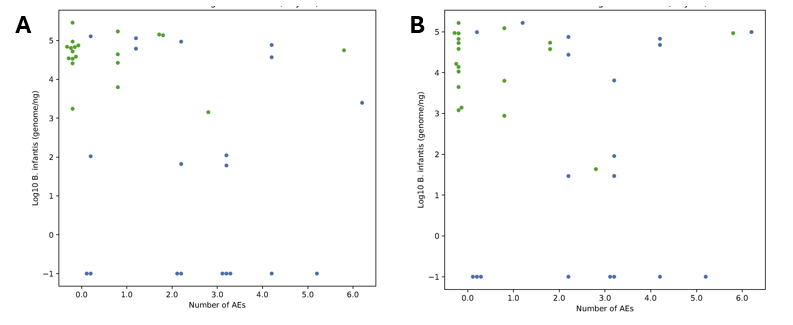** |

| Supplementary Figure 5: Associations between FCT classification and CONSTELLATION outcomes. (A) Scatterplot showing change in WAZ from day 1 to day 56 stratified by FCT classification at day 56 (FCT1/3 vs. FCT2). Horizontal bars indicate group medians. (B) Scatterplot indicating the cumulative number of adverse events (AEs) reported through day 90 for infants classified as FCT1/3 vs. FCT2 at day 56. *p=0.03 |
| --- |
| **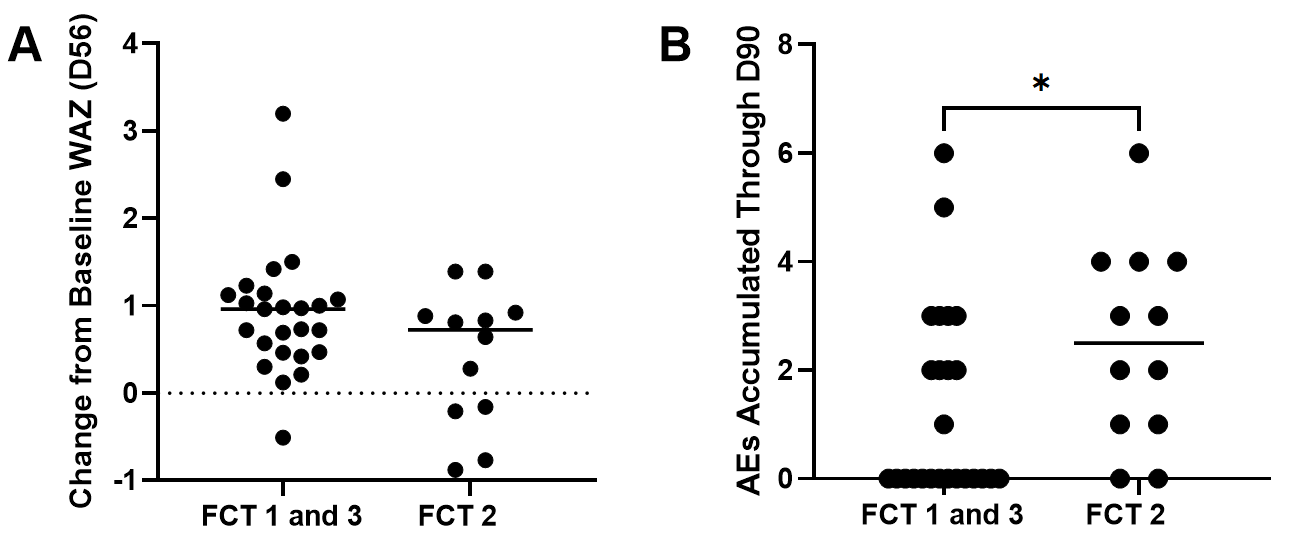** |
